# From Dissidence to Heroism: Constructing an Ideal Post-Communist Identity in the Czech Republic

**DOI:** 10.1177/08883254231168409

**Published:** 2024-11-01

**Authors:** Muriel Blaive

**Affiliations:** University of Graz, Graz, Austria

**Keywords:** post-Communism, memory politics, historical narrative, anti-Communism, remembrance as ideology, heroization of social actors

## Abstract

Post-Communist memory politics has occupied a highly disputed symbolic position ever since the Velvet Revolution in Czechoslovakia. This article presents the case of Czech student leaders of the revolution, especially Monika Pajerová (since 2002 Monika MacDonagh-Pajerová), who co-organized the 17 November 1989 demonstration that initiated the fall of the Communist regime. It focuses on the social and political movement “Thank You and Goodbye!” (“Děkujeme, odejděte!”) organized by the same students in 1999. The article analyzes this particular moment as a turning point in post-Communist development: the students’ genuine concerns and their sincere analysis of the democrats’ own shortcomings in and after 1989 created the background for a new ideology of anti-Communist remembrance that would become prevalent in the Czech public sphere in the 2010s. The post-Communist regime’s refusal to integrate the Communist period as a legitimate part of national history prevented the building of an appeased democratic society. It was the original sin of the post-Communist regime, one that would create the need to rewrite the national script concerning Communist history.

There were relatively few dissidents in Czechoslovakia before 1989: fewer than 2,000 people signed the Charter 77 manifesto, for instance. After Communism’s fall in 1989, a number of these signatories chose not to capitalize on what was now considered a prestigious gesture, and they disappeared into private life. But others aspired to belong to the post-1989 elite, and they raised social capital by highlighting their previous engagement against the Communist regime.

This spontaneous association between dissent and social capital was progressively extended as decades passed, and it came to include ever wider social groups, such as the cultural underground of the 1970s and 1980s and the anti-Communist resistance of the 1950s. Within a generation, dissent was in fact replaced by heroism as the ideal societal representation of the relationship to the Communist past in the Czech Republic. Testifying to this replacement is, for instance, law 262/2011, on “Participants in Anti-Communist Opposition and Resistance,” which sought to identify and reward almost any form of opposition to the regime. Victims condemned on the basis of law 231/1948, on the “Protection of the People’s Democratic Republic”—an act that had served as the legal basis for Communist repression—had previously been rehabilitated on the basis of law 119/1990, “On Court Rehabilitation.” Law 198/1993, “On the Illegality of the Communist Regime and on Resistance to It,” had then sanctified anti-Communism as a core value of the new, post-Communist regime, calling the former Communist Party of Czechoslovakia a “criminal and reprehensible organization.” Now, in 2011, many more people could apply for what became informally recognized as the status of legitimate hero.^1^

How did the ideal Czech societal status evolve from victimhood and dissent to heroism? I present as an example in this article the case of a few Czech students, especially Monika Pajerová (since 2002 MacDonagh-Pajerová), who was among the organizers of the student demonstration on 17 November 1989 that initiated the downfall of the Communist regime.

I will endeavor to show that the social and political movement “Thank You and Goodbye!” (“Děkujeme, odejděte!”), organized by these same students ten years later, in 1999, was a turning point in the post-1989 regime, one that would transform genuine concerns and a sincere analysis of the democrats’ own shortcomings into a new ideology of anti-Communist remembrance, which would become prevalent in subsequent decades. The refusal to introduce any nation-wide form of reconciliation concerning the Communist past prevented the build-up of an appeased democratic society. It was the original sin of the post-Communist regime, which would, in time, create the need to rewrite history so as to delegitimize the place of Communism in national history. I use as main sources for analyzing the construction of this public narrative media articles and talk shows, as well as memoirs and interviews, because classic historical studies of the post-1989 period, which would “take into account the lived experience of social actors,” are still rare.^2^

## Personal Memories and Collective Autobiographies: A Contrast

From September 1990 to September 1992, I was enrolled as a student in the Faculty of Czech at the University of Oriental Languages in Paris while studying at Sciences Po. I was already working on Czech history, and I followed the cultural activities of the Czechoslovak embassy in Paris. A new Czechoslovak ambassador, Jaroslav Šedivý, and a new cultural attaché, Monika Pajerová, had been appointed. Šedivý was a jovial personality, and at what seemed every event organized until 1992 (when I left Paris for Prague), he recalled how he had inspired Kundera to develop his character Tomáš in *The Unbearable Lightness of Being*—“minus the philandering, as I keep repeating to my skeptical wife,” he would tell audiences again and again to their unfailing mirth.

Another anecdote Šedivý was fond of repeating was that his young cultural attaché, Monika Pajerová, had been “one of the leaders of the Velvet Revolution” in November–December 1989. But Pajerová was very shy, and she unfailingly blushed bright red to the roots of her very blond hair. She protested every time that she was no leader, she just happened to be there and to speak foreign languages, and people able to communicate in English and French to the outside world were needed in these circumstances.

Twenty years later, the impromptu press attaché of 1989 had discovered her inner heroic identity after all. A 2009 billboard installed on Prague’s Wenceslas Square to celebrate the twentieth anniversary of the Velvet Revolution stated (in Czech and English):

Monika Pajerová was among the top personalities of the student generation who managed to lay the ground of the independent student movement during the mid ’1980s.[. . .] During these unsettled days when the communist regime was collapsing, Monika Pajerová organized the strike committee at the Faculty of Arts and also functioned as a spokeswoman of the University Strike Committee and later also participated in the newly established Civic Forum activity.^3^

The author or authors who had drawn up this billboard on behalf of the NGO Paměť národa (Memory of the Nation), under the patronage of the Institute for the Study of Totalitarian Regimes, quoted Pajerová as saying (the clumsy English grammar is their own):

We created the STIS [Studentské tiskové a informační středisko] (Student Press and Information Center), where all the editors and fellow colleagues used to meet on Thursdays. We discussed there what each editor will publish, what exhibition we’ll do. We also used to invite people who weren’t supposed to exist—signers of Charter 77, people from VONS [Výbor na obranu nespravedlivě stíhaných] (Committee for the Defense of the Unjustly Persecuted [. . .]) or people from the Independent Peace Organization. We signed the “Několik vět” (A Few Words—written by Václav Havel) document too and then we organized the demonstration on November 17th 1989.^4^

The Institute for the Study of Totalitarian Regimes, which had started to operate one year previously, in 2008, was the main sponsor of this action. Its founder and director, Pavel Žáček, was another former 1989 student. In fact, according to this 2009 billboard narrative, both Pajerová and Žáček were leaders of the Velvet Revolution. The billboard now revealed the revolution was only one of the many actions they had planned. The English translation provided on the panel claims: “This really conspiring group never published anything of course. Its task was to establish a very powerful network throughout universities.” After STIS united with another movement, Stuha, led by Šimon Pánek, an

unbelievably sophisticated organization of future strike committees was created throughout the faculties. Its ability to act quickly and its professionalism surprised us. It is probably an irony of fate that the November 17th demonstration was only one of the many planned activities which we prepared together. But the other ones were swept away by the avalanche of changes that came after the police raid on National Avenue.^5^

After 1989, Pajerová worked in public service as a diplomat, first in Paris at the Czechoslovak/Czech embassy, then in Strasbourg at the Council of Europe. She became active again in the Czech public sphere on the occasion, and in the aftermath, of the 1999 commemoration of the Velvet Revolution. She created an NGO called Ano pro Evropu! (Yes to Europe!), she dabbled in politics, and she hosted a talk show on Czech television, on top of becoming a lecturer at New York University in Prague. This article argues that her role during the Velvet Revolution was redefined alongside her blooming career: at first, she was depicted and depicted herself as a modest cog in the wheels who happened to be in the right place at the right time in 1989 (the narrative that I heard in 1990, provided that my own memory is reliable), but was otherwise a loyal citizen of the Communist state who enjoyed the privilege not only of studying in Czechoslovakia but of repeatedly travelling, and even studying, in the West, as she explains herself in her memoirs; only later did she “become” one of the genuine student leaders of a movement that was, we were now told, actively preparing to bring down the Communist regime.^6^

How did this transformation occur? I focus in this article on a major stage in this development, which is the year 1999 and the tenth-anniversary commemoration of the 1989 Velvet Revolution.

## The Public Proved to Be Skeptical about the Achievements of the Revolution

In general, the narrative that the students and new political elites promoted after 1989 was one of rupture. It refused to acknowledge continuities between the new democracy and the pre-1989 regime. This refusal put the new elites in an awkward situation vis-à-vis the wider public, which had, on the contrary, no qualms acknowledging and underlining such continuities. The media abundantly documented this last trait. For the tenth anniversary of the revolution in November 1999, for instance, Czech public radio prepared a report that featured a number of street interviews. This report distinctly shows that the public did not seem as impressed with the achievements of the revolution as the new regime might have wished, either on the economic and social levels, or as far as human dignity was concerned. Here is how the proverbial “ordinary people” answered the question, “Have we succeeded in the past ten years to deal with the Communist past?”^7^

“Well, I haven’t felt anything much myself, my parents live in southern Bohemia, they work in agriculture, it’s still the same there, the regime doesn’t affect agriculture, I think, it’s still the same, the same hard work, and nobody gives anyone anything for free.”^8^ “I think we weren’t doing badly under the Communists and we didn’t have what we have now, this horrible Sherwood park at the Prague Central Station where we are standing right now.”^9^ “We have not come to terms with the Communist past, I fundamentally think we haven’t. The economy of the country today is controlled by the Communists, absolutely nothing was done against anyone, yet it would have been easy to document the crimes.”^10^ “I don’t see that we are any better off than we were before, not me personally, anyway. As for myself, I’ve had three heart attacks and the Communists gave me a pension and the new people in power now took it away. And so I actually have to go to work even though I had three heart attacks.”^11^ “The people who were in the Communist party and who were in power just turned their coats.”^12^ “Listen, I am an amputee and if I die here on the pavement, everyone will shit on me—this would not have happened under the Communists.”^13^ “What did we do this democracy for, what is it good for, who shook their keys? I didn’t.”^14^

Live reactions from members of the audience who phoned in on the program accompanied these pre-recorded street interviews: “My answer to your question is no, we haven’t [successfully dealt with the past], because we haven’t wanted to,” said the first listener.^15^

Dealing with the past would have entailed dealing with ourselves in the vast majority of cases. Well, yesterday and the day before, when I watched the TV documentaries, [. . .] it was clearly visible that ten years ago one didn’t understand what was going on, but one was actually happy because one had his tripe soup, goulash, 12° beer, time clock, cigarettes, newspapers. And even if we look at the electoral preferences today, it seems to me that democracy in this country is only valued by some 15%, maybe 20% of the citizens, and this only if I am very optimistic. So we haven’t dealt with it, because it wasn’t possible in such a short time, especially not since the nomenklatura cadres became the new political elites, but also due to the fact that we were a grey army of mice.^16^

The reaction of surprise and shock of the talk-show guests, the former dissident and now politician Rudolf Battěk and the former student leader Monika Pajerová, was enlightening as to the disconnect between the new elites and part of the population. Battěk commented,

These are bitter words, and one is at a loss how to respond [. . .]. Part of the historical tragedy is in the way it all happened ten years ago [. . .] if there hadn’t been those post-Communists who were sort of holding it back, seeing in those full Communists [*v těch plnokomunistech*] their former brothers, and still sort of toning it down, then maybe we could have done more legislative work to prevent the chance of outright fraud, scamming, and asset-stripping. But it will mainly take time and a generational shift for the remnants of Communism to somehow burn themselves out and for new people to come in.^17^

Pajerová seconded the idea that there were more positives than negatives in the post-1989 regime and that democracy and human rights were a success. However, she admitted, the economic transformation had “left several people on the side of the road,” which manifested itself as “frustration within society.” Even more of a problem was, according to her, a “loss of faith in societal development,” a “lack of trust in the political elites,” and the “feeling of citizens that their fate is decided elsewhere.”^18^

Battěk lamented that his generation had not been “morally prepared” for the change; he and his peers “had to take on board some of these ex-Communists, those who indicated their willingness to help, and many behaved decently,” but others were a “bunch of crooks.” Pajerová concluded,

[The Communists] had everything, the army, the police, the secret police, agents, a network of informants. The power was ready, whereas we only had our bare hands, we had nothing more than public opinion on our side. So, we did not have any other choice but to try and avoid spilling blood, avoid that people kill each other.^19^

Pajerová’s arguments are not without merit. As I have argued in another article, the dissidents suffered from a lack of legitimacy in the revolutionary days.^20^ Their movement constituted the only alternative to Communist power, but its members represented only a tiny segment of society. Havel surfed on a thin wave between pushing for a radical change, gaining sufficient legitimacy to convince a majority of society to support him, defeating his rival Alexander Dubček for the presidency, and avoiding violence. The result was positive insofar as the revolution succeeded, and it did so without violence, but it also sowed the seeds of future dissatisfaction.

## Whose Fault Was It If the Revolution Partly Failed?

The oral historians Miroslav Vaněk and Pavel Mücke confirm, after having interviewed hundreds of ordinary citizens, that “[r]eferences to inequitable conditions, stealing, and corruption are a leitmotif of the recorded interviews. ‘Well, to put it bluntly [. . .] assets were simply pillaged.’”^21^ The privatizations of the 1990s remain a particularly sore point:

We have heard dozens of similar complaints about Czech privatization in our interviews.[. . .] It was impossible for [. . . people] not to hear Václav Havel’s words of warning, when he described the economic system of the late 1990s as “mafia capitalism.”^22^

If almost everyone agrees that the revolution was far from perfect and the post-revolutionary period started on the wrong foot, conservative pundits have generally blamed it on insufficient lustration of former Communists—lustration being the exclusion from public service of any citizen with who before the revolution was a secret police agent or collaborator, or a high-ranking Communist Party functionary. Yet I would argue that the Czech lustration policy was both too harsh and misplaced. It excluded people from the administration arbitrarily, including many competent administrators, but this harshness deflected the need for action. The Czech state adopted only collective measures in its endeavor to deal with the Communist past—collective lustration, collective rehabilitations, and a collective moral indictment of all former Communist party members—while refraining from establishing personal responsibilities.^23^

By so doing, it pushed the former apparatchiks toward the economic sphere and left it largely at their mercy. These apparatchiks were the only individuals who had sufficient social, economic, and human capital after 1989 to invest in the new economy. Soon they were perceived as having captured a large part of the national wealth in a climate of “non-transparent privatization processes, clientelistic networks, and the export of profits abroad,” all while a public narrative on “winners and losers” was taking shape.^24^ As both Battěk and Pajerová readily admitted in the radio talk show in 1999, neither the dissidents, nor the students, nor any other part of the rather loose Czechoslovak opposition, nor the West, for that matter, had been prepared for such a radical change.^25^ Nor could they have been. When Václav Klaus appeared on the political stage in 1989, the former dissidents could feel only relief despite the personal misgivings of Václav Havel, as they had little to no idea how to manage the economy.^26^ They also did not have any real understanding in the subsequent years of what Klaus really accomplished as finance minister—which amounted, with hindsight, to little less than selling the country off to newly established barons of industry and incrementally destroying the core value of Czech society at the time, egalitarianism.^27^

For a perspective from everyday life, the engaged (anti-Klaus) novelist Michal Viewegh wrote the novel *Bliss Was It in Bohemia with Klaus*, in which he chronicled the transformation years from 1989 to 2001. One scene is particularly telling: the author’s father, who was a member of Civic Forum, supported an old friend, Zvára, when the latter applied in 1991 to become director of the firm where the author’s father had worked for the past quarter-century. This support was surprising because the two had parted ways after 1968, when Zvára had become a Communist and made his career under normalization, while Viewegh’s father had refused to compromise himself and thus remained a simple worker. But the father now supported Zvára in the name of economic efficiency and because “we are not like them.” The result was that Zvára, once appointed, nominated only “his” people (other former Communists) to key positions, and when the father protested, he was fired. As an added twist, he was purposely fired on the day preceding his fiftieth birthday, in the midst of an economic crisis—a circumstance that broke him.^28^

The journalist and academic Jan Čulík was also fired from the Czech section of Radio Free Europe when he tried to report on the way the same Václav Klaus, prime minister since 1992, was receiving private honoraria (one million crowns in 1994) for holding public speeches while in office.^29^ Klaus went on to replace Havel as president in 2003, but widespread asset-stripping rumors nearly caught up with him when the Czech Senate impeached him and charged him with high treason in the very last days of his second mandate in 2013. He was accused of amnestying, as president, a number of “high-profile corruption cases—cases that involve[d] millions of dollars in asset-stripping, bribes and fraud.”^30^ The charge was dismissed by the Constitutional Court on procedural grounds (Klaus was no longer president when the case came before the Court), but this episode testifies to the exasperation of Klaus’s political opponents and certainly of a part of public opinion. A reading of the contemporary press shows there was already in 1999 an acute sense in the Czech public sphere that the economy of the country had been confiscated by the former apparatchiks with the blessing of the post-1989 government and parliament. The above-mentioned radio talk show, indeed, concluded by quoting an article by Petr Pavlovský in the daily newspaper *Metro*:

Passing [. . .] laws that have enabled large-scale robbery of the state and of the population is the topic at hand.[ . . .] One last question remains, will we at least know who lent how much and to whom, since we paid, and will have to pay for it?^31^

The main issues that agitated the Czech public sphere in 1999 were thus all present in this radio talk show: the disappointment of the public concerning the economic transition; their doubts concerning the integrity of the new elites, a diffuse feeling of betrayal of the democratic ideals of the Velvet Revolution, the surprise of actors of the Velvet Revolution at the virulence of these complaints, and their awareness that their moral and political guidance was needed and expected more than ever. In consequence, former student leaders decided to take action. A petition, which later expanded into a demonstration and metamorphosed into the political movement “Thank You and Goodbye!” was organized by seven former student leaders of 1989: Josef Brož, Igor Chaun, Vlastimil Ježek, Martin Mejstřík, Monika Pajerová, Šimon Pánek, and Vratislav Řehák. Their manifesto demanded the resignation of two political leaders: Václav Klaus (then president of the lower chamber of Parliament) and Miloš Zeman (then prime minister). It further demanded the end of the so-called “opposition agreement” that these two politicians had concluded as well as the restoration of “civic decency.” The petition garnered 200,000 signatures within a few months and was, according to opinion polls, supported by 56 percent of the population.^32^ The public was evidently hoping that the former student leaders would emerge as a new political force that would lead the country on a renewed crusade for honesty and decency—in other words, that they would revive the ideals of 1989.

## From a Collective Diagnosis of Partial Failure to the Failure of Individual and Collective Mobilization

Monika Pajerová’s analysis of the situation in 1999 appears sincere and lucid. On another talk show at the end of the year, this time on Czech Television, she declared,

At the time [in 1989], we did not assume that the Communists would somehow return to the political stage. We just wanted to talk about how we were all going to live in this country. And I think that today we are in danger of a very serious thing happening, if it has not already happened, namely that ordinary citizens have the feeling that politics is again being led somewhere outside of their reach, and we are even witnessing the fact that, somehow, the political and economic spheres are intermingling. The former leaders of the Communist economy have become the biggest capitalists, they sponsor the biggest political parties, play tennis with them [Václav Klaus was famous for his tennis practice] and go to various parties, breakfasts, and dinners, thus creating a completely homogeneous [. . .] layer of people who simply help each other out in the economic and political sphere. Then there is a big gap, in which there is nothing and nobody, and then there are the rest of us, the so-called civil society, who have no choice but to be silent, go to work, not grumble too much, and go to the polls every four years and choose from the few parties that exist here, but most people will say, “I don’t know who to vote for, I don’t trust any of these parties at all.” [. . . A] tragedy will arise when the gap widens so much that the citizen will no longer even want to go to the polls [. . .] look at how many members a political party must have, and the Social Democratic Party has maybe 13–14,000 of them—who does it even represent?^33^

The journalist Tomáš Brzobohatý quoted Jakub Patočka, editor-in-chief of *Literární noviny*, a liberal cultural newspaper, who went so far as to describe the Velvet Revolution as a “revolution of missed opportunities.” “We are living again in an atmosphere of normalization. We don’t speak to elected politicians, but in return for our silence we receive benefits from them.”^34^

Unsurprisingly, however, the two political leaders targeted by the students’ petition did not agree to step down or even admit that the situation was critical in any way. As Prime Minister Miloš Zeman put it, why should they, who were legitimately elected and re-elected, resign as the result of a public appeal issued by a few students? He remarked not without humor that there had been only 2,000 people at the original demonstration on 17 November 1989, but the number of revolutionaries appeared to increase exponentially with each passing year.^35^ Meanwhile, Klaus’s advisor Ladislav Jakl condemned the students’ appeal as a “fundamental attack on democracy in our country.”^36^ Klaus, who was then leader of the opposition, even attempted to delegitimize one of the student leaders, Šimon Pánek; he asked what Pánek, who had just spent time in Nagorno-Karabakh on a humanitarian mission, knew of the current situation in the Czech Republic.^37^ He claimed that “such cries ha[d] no political weight.”^38^

Václav Klaus professed to have been neither surprised nor disappointed by the development of the 1990s: “I knew we would not create paradise on earth,” he said.^39^ He was not alone. The main daily at the time, the conservative *Lidové noviny*, collected the impressions of several other major political actors:

“I was not disappointed by the post–Cold War development. I had a fairly realistic picture of where we were,” says Petr Pithart, the post-November prime minister of the Czech government (1990–1992), now deputy chairman of the Senate. “Despite all the shortcomings, I cannot be disappointed,” rejoins Jan Ruml, the former interior minister and now head of the party Civic Union. His father, the publicist Jiří Ruml, praises the freedom of speech that Czechs have gained. “I was pleased that the transformation has begun successfully. I regret that it started to lose momentum and stopped,” said Jiří Ruml. “I am glad that we have democracy. But I am sorry that it is being abused,” said the former deputy prime minister Valtr Komárek, who is now retired.^40^

As in 1989, the former students toyed with the idea of getting involved in the political sphere, but half decided against it, and the rest failed to create any meaningful political party. They did want Klaus and Zeman to resign; as underlined above, the petition “Thank You and Goodbye!” stemmed from their indignation at seeing the two main political parties, the conservative Civic Democratic Party and the Social Democratic Party, adopt a so-called “opposition agreement,” which essentially silenced any opposition to government policy and stifled democratic debate. But perhaps the most visible symbol of the continuity in behavior, if not in policy, of the post-1989 leaders was, as the priest and former dissident Tomáš Halík recollected, the gigantic electoral billboard of Václav Klaus that took the place of the former statue of Stalin in 1998. Erected in 1955, the statue had been the biggest Stalin sculpture in the world. It was destroyed in 1962 in the frame of de-Stalinization. To replace it by the Klaus billboard gave a megalomaniac impression that was at odds with the message it purported to convey: “We think differently.”^41^

Igor Chaun, another former student leader, criticized both Klaus and Zeman, the leaders of these two main parties, for their disregard of the population. Czechs were supposed to elect them every four years but otherwise endorse their actions with “the passivity of robots.”^42^ The symbolic dimension of the moment was strong: as Pajerová put it, “We are standing at a crossroads, the future of the country is at stake. We must decide if we are going back to the East or together back to Europe.”^43^ As for herself, she had chosen her camp: she had been a diplomat in Strasbourg at the Council of Europe between 1994 and 1998, and she would, shortly after this petition was launched, create a new NGO called Yes to Europe! which would promote the entry of the Czech Republic into the European Union. Jan Hartl, a sociologist and director of the polling institute STEM (Středisko empirických výzkumů, Institute of Empirical Research), emphasized this cultural dimension, too: to “belong to the developed West,” Czech society had to prove that it was “not passive,” that it was “not renouncing” the building of democracy, and that it was “capable of expressing a certain anger” in the face of politicians who were “to a certain extent disconnected from the real problems of this country.”^44^

Shortly before Christmas 1999, the seven signatories of “Thank You and Goodbye!” went on a surprise visit to their elected officials. Even though the legislators were supposed to hold office hours every Monday, fifteen out of the 22 MPs representing the Prague region in the lower house of Parliament were unavailable; three had their phones busy for several hours, and one had changed his phone number and made the new one unavailable. Only three MPs could be found in their offices: one received the activists without reservation, one (Klaus) agreed to speak only with three out of the seven visitors, and one refused to speak to them and fled to escape what he called “intellectual terror.” In Brno, the situation was comparable: of the thirteen legislators who were supposed to be available for public consultation, only three were physically present.^45^

Chaun and the other authors of “Thank You and Goodbye!” had reason to find the attitude of both Klaus and Zeman contemptuous toward them, hence a logical response would have been to compete against them for political mandates. They indeed toyed with the idea: “It turns out that there is a great social demand for a new political party,” said Chaun.

The polls show it, and the letters we get show it, too. But we’re also reminding people through this action that Mr. Klaus is wrong: we don’t have to start a political party to make our voices heard. [. . .] However, the demand for a new political party is strong, so it is likely that one will be created amidst some of our signatories.^46^

Vlastimil Ježek, another one of the student leaders, who had meanwhile become the director of Czech Radio, confirmed that “a certain political platform” would probably come out of this movement.^47^

However, when it came to putting these preliminary intentions into practice, Ježek decided not to involve himself otherwise than by occupying a junior, managerial position. His “embedded distrust in political parties” was “too strong,” even though he admitted that “no one had invented a better system to date.”^48^ Igor Chaun “supposed” that a new political subject would be born, which would work together with the newly formed Information and Coordination Center of Regional Activities and Civic Forums.^49^ However, the representative of said Coordination Center, Petr Koura, stated that the Center would dissolve if a new party were created.^50^ Chaun ultimately decided to remain a filmmaker rather than involve himself in politics. The independent senator Václav Fischer, tipped as the potential head of a future party, refused even to meet with the students.^51^ As far as the other signatories were concerned, Šimon Pánek decided to continue doing humanitarian work. Martin Mejstřík did run for the Senate in 2002 and got elected, while Josef Brož and Monika Pajerová were involved in creating a small party, Cesta změny (Way of Change), which enjoyed little success. Pajerová also professed a vague aim to establish a federation of civic movements, but this project never came to fruition, although she did become the chair of yet another small party, Naděje (Hope), which proved only marginally more attractive to voters than Cesta změny.^52^ The fact that so many of the former students decided to refrain from engagement in institutional politics after 1999, while others (apart from Mejstřík) failed in their efforts, shows their unwillingness or inability to nurture not only a new party but civil society more generally, even though both they and the wider public were aware that it was needed. The opportunity came and went.

## The “Original Sin”: The Exclusion of Communists as Dialogue Partners

During a debate organized at the Faculty of Arts, the new students of 1999 complained that the old students of 1989 had not been “harsh enough” with the Communists during and after the Velvet Revolution. Where Havel had been inclined to blame citizens themselves for the long spell of passivity and depression that characterized the 1969–1989 normalization period—a narrative that he supported both before 1989, during the 1989 events, and in his first speeches as newly elected president—by 1999 the public mood of self-flagellation had given way to frustration with the progress of the post-1989 transition.^^53^^ The fact that the 1989 exit had been negotiated with the Communist authorities had apparently, by then, been almost completely forgotten. Culprits were eagerly sought, and both “the Communists” and the “opposition agreement” were designated as such. In subsequent years, after the opposition agreement collapsed, “the Communists” remained a target of elites claiming to represent people frustrated by the transition.

The former student leaders cannot be held responsible for the imperfections of the transition out of Communism. However, their lack of action between 1990 and 1999 contributed to worsening the situation. Monika Pajerová was fully aware of the fact that the slow development of Czech civic society was a major handicap in the postCommunist transition. She emphasized that a society in which there was no dialogue was one that did not foster the development of a civic spirit. She called for a broad and inclusive social dialogue that would restore one of the ideals of the revolution: dialogue.^54^ As she had stated in her opening speech at the demonstration of 17 November 1989, “It is all about dialogue, because there is more that unites us than divides us.”^55^ In 1998, she described to the oral historian Miroslav Vaněk the reaction of the public to her speech in 1989:

People clapped a lot because it was the feeling of probably each and every one of us that nobody had a completely pure conscience, everybody got a little mixed up with the regime, everybody was taking the exams in Marxism-Leninism, but what we had in common was a bigger part, and that is what we had to build upon.^56^

However, only one year later, in 1999, her narrative became substantially different: she had now seemingly forgotten that her call for dialogue in 1989 was one of dialogue *with* the Communists, not *against* them. If the devil hides in the detail, it is worth pointing out that, by 1999, the former students’ notion of “dialogue” had evolved, and it now actively excluded Communists and the part of the electorate who voted for them. As Igor Chaun put it in early December, “We invite everyone to join us for a decent and peaceful meeting [on 3 December 1999]. Our slogan is: Take your Christmas bells with you, but as to you, Communists: stay home.”^57^ Monika Pajerová doubled down on the exclusion of Communists:

I think the people who run the media should exercise judgment in the sense that they simply shouldn’t invite a [. . . Communist leader] like Miroslav Štěpán to the most watched debate at noon on Sunday. For one thing, it spoils our appetite, and for another, I think these people should simply be marginalized by any means possible. This means not inviting them to television, not inviting them to the radio, not interviewing them [. . .] It sounds terribly black and white, but I stand by it, I would just push them out of the public sphere and out of the mass media [. . .]. That’s why I appeal to the judgment and reason of every editor, every responsible person who organizes such a debate.^58^

However, if the former students’ notion of dialogue socially cancelled both the elected politicians, such as Klaus and Zeman, as well as the past and present Communists, we might wonder who their potential discussion partners would have been. Between whom and whom should a “dialogue” have then taken place? The looming danger was rather one of monologue.

## From Excluding Communists to Rewriting History

Interestingly, one of the most reflective responses to the 1999 demonstration and speeches of the former student leaders, even if permeated by ideological language, can be found in the Communist-sympathizing and rather low-brow periodical *Haló noviny*. It quoted the narrative established among new elites, including the former student leaders—a narrative that blamed all manner of present difficulties on Communists:

“Ten years after we embarked on the struggle for democracy, the Communists receive 20% of the votes, society is disillusioned, and instead of dialogue we are witnessing only a series of monologues,” appealed Monika Pajerová, who is now a diplomat. “We are where we almost inevitably had to end up because we are too afflicted and infected by the past,” said Pavel Žáček, former editor-in-chief of *Studentské listy* [Student Journal].^59^

Yet *Haló noviny* was one of the rare outlets to notice a difference in tone among the former student leaders. Being attuned to the popular frustrations caused by the economic and political transition, the newspaper was quick to notice that, in some, frustrations with the policy of dealing with the past were related to frustrations of dealing with the present. The article concluded,

“The slogan ‘*We are not like them!’* was important, but unfortunately it seems to me that the current leaders sometimes behave in an even worse way.^60^ We blamed power and privilege, but the situation hasn’t changed much. As for coming to terms with Communism, we have neglected it,” opined Martin Mejstřík, perhaps the most critical voice on the podium. Although some of today’s students did not “step out of line,” and they did ask why there were no cadre commissions after November and why the Communist legacy was not treated like Nazism, some of the questions and speeches at least hinted at some critical thinking.^61^

Many 1999 students and other citizens were in favor of resorting to radical measures to get rid of former Communist powerholders. Yet, in effect, the former students replicated in their rejection of the Communists the methods that had been standard practice in Communist Czechoslovakia against the enemies of Communism: rejection, boycott, and “intellectual terror.” Part of this radicalization had to do with the fact that the Communist Party of Bohemia and Moravia had by 1999 become the third most popular party in the country, and it would receive 18.5 percent of the vote at the parliamentary elections in 2002. With the “opposition agreement” between the main parties, the Communists threatened to become the backbone of the opposition. This is why we can interpret the movement around “Thank You and Goodbye!” as a turning point in the post-1989 regime. From genuine concerns and a sincere analysis of the democrats’ own shortcomings in and after 1989, the movement metamorphosed into a new ideology of anti-Communist remembrance that would gain extraordinary importance in the following decades.

Indeed, a prescient journalist, who signed only with the letters DV, gave birth in 1999, perhaps offhandedly, to one of the most powerful arguments of the subsequent anti-Communist movement:

Šimon Pánek, Monika Pajerová, Martin Mejstřík and other former students who started the revolution were not afraid to speak out against the Communist regime at the time. What are their feelings today, when they learn from the media that the Communist Party of Bohemia and Moravia is the second strongest party in the Czech Republic? We must not allow for people who experienced Communism to forget what happened here. We, the young, hardly remember what the situation was like in this country.^62^

Here was the warning: if one did not constantly remember the evils of real Communism, the Communist Party would inevitably come back to power. Indeed, the opening line of law 181/2007, which created the Institute for the Study of Totalitarian Regimes, was, “Those who do not know their past are doomed to repeat it.”^63^

An unsolvable paradox of the Velvet Revolution is that it claimed to foster a new way of making politics but was accomplished in the name of “anti-politics” by social actors who were themselves reluctant to get involved in politics. As a result, those among the revolutionaries who genuinely were interested in politics, first and foremost Václav Klaus, had the way wide open to them. As in the 1950s, the Czechs and Slovaks wished for Communism to fall and the transition to proceed without them having to contribute much, if at all. Back in the 1950s, it had been “the Americans” who were pegged to come and free Czechoslovakia, whereas in 1989, it was “democracy” and “the market.” ^64^ But the effortlessness of the process invoked by popular imagination was remarkably similar.

Under the guise of “never forgetting,” and in a context defined by a sense of powerlessness and a lack of desire to get personally involved in politics, some of the former students, including Monika MacDonagh-Pajerová, were destined in the first decade of the new millenium to transform their pertinent analyses of 1989 and 1999 into a powerful anti-Communist ideology, a narrative that would compensate for their actions’ failure to achieve the desired results. They would soon endeavor to blame many present woes on past Communists, and it could not have been otherwise, insofar as the ideals of 1989 were far from being fully delivered by the post-Communist regime. The democracy and prosperity promised in 1989 materialized in the guise of corruption at the highest level, the extreme enrichment of a few, and the economic improvement of many, but also the economic stagnation of vast parts of society and their disenfranchisement from politics.^65^ Not only did the new democracy, which was assumed to be superior to Communism, fail to eradicate Communism, but at the end of the 1990s, the Communist Party of Bohemia and Moravia was still strong.

This is how we arrive at the difference between 1990 and 2009 in Monika MacDonagh-Pajerová’s biographical self-stylization that I described in the incipit of this article. The more difficult but also more beneficial path to solve the contradictions of the Velvet Revolution would have been for members of society to face their own pasts, their own involvement, and their own behavior, and it would have been for intellectual elites to provide leadership and guidance in this painful process. Václav Havel did suggest such a path after 1989, but he abandoned the attempt soon enough—it was doubtless too demanding at a time when energies were mobilized toward the more urgent economic transition out of Communism. From the moment Monika MacDonagh-Pajerová described the Velvet Revolution and its subsequent development as principally good and justified, and from the moment she, by so doing, delegitimized the frustration and skepticism of the Czech public as expressed in 1999, the former students could only promote their own narrative with ever greater radicalism in an effort to align it more closely with the unhappy reality of the country: in this narrative, it was the Communist regime, and only the Communist regime, that had been principally “bad.”

## Conclusion

Has Jaroslav Šedivý’s 1990 quip that Monika Pajerová was a heroine of the revolution become the new truth? The answer is that the “humble” and the “heroic” interpretations of Monika MacDonagh-Pajerová’s role in 1989 are equally true, insofar as the notion of historical truth is a weak concept that changes with time and is inscribed in a constantly evolving context. Her example, and the cases of the other former students, shows that their personal trajectories and the rewriting of their biographies has progressed in connection with the tenets of ideal conduct as they were redefined over the years by the official memory politics of the Czech state as well as by societal evolution of the relationship to the Communist past. In 1990, the students were modest participants dedicated to the collective success of the newly established democracy; in 1999, Monika Pajerová had already become, in the journalist Erik Tabery’s words, a “legendary figure of the student movement.”^66^ By 2009, those among the post-1989 elites who had a career or a reputation at stake embraced the call for heroes that state institutions promoted to divert attention away from the failures of the de-Communization policy—that is, the dismantlement of the welfare state and the impoverishment of large swathes of society.

After 1999, the country found itself in a deleterious form of post-Communism. The politicians whom the students called upon to resign did not resign, and no new party of consequence was established. Havel endorsed the students’ appeal as if he had not been a leader of the post-1989 development himself, thereby shirking his own degree of responsibility.^67^ The former student leaders became more radical. But the generation of political activists that would come next, personalities who had to reinvent such a form of legitimacy for themselves outright, would become even more intransigent.

The refusal to engage in a “dialogue” with Communists stemmed from the refusal on the part of the post-1989 political and intellectual elites to integrate the Communist period into legitimate national history. I call this the “original sin” of the post-Communist regime. By externalizing the Communist period as an era that did not truly belong to Czech history, and by pretending that Communism was alien to Czech culture, the new democracy adamantly rejected any possibility of national reconciliation and maneuvered itself into the position of excluding a substantial part of the electorate, at least until the Communist Party of Bohemia and Moravia died its natural death in the 2010s. This exclusion might have appeared politically legitimate, but it greatly contributed to creating a feeling of disenfranchisement for a large part of the left-wing electorate, which subsequently stopped voting.^68^ Moreover, to blame “the past” while pretending that the new political actors were immune from it was neither credible nor constructive.

The adamant refusal to amend this anti-Communist posture and its further radicalization eventually created an ever greater need to rewrite history so as to blame solely “the Communists.” At the beginning of the 1990s, a certain category of the population (“collaborators with the secret police”) had been designated as traitors to the nation. As this designation could not justify the shortcomings of the transition, the post-1989 failings were then blamed on “the Communists,” past and present. The past Communist regime was represented in increasingly hyperbolic terms, while those who had opposed Communism were placed on an ever higher pedestal. In 1989, it was the dissidents who held the moral high ground; in 1999, it was the turn of the “students”; in 2011, it would become the “resistance.” The ideal post-Communist identity has thus evolved, and it will doubtless continue to evolve in the coming years.

## Notes

1. Veronika Pehe has reflected at length on the societal evolution from “petty heroism” in the first years after 1989 as an ideal representation of the Communist past in Czech culture to a more dramatic search for “true” or “legitimate heroism” in the 2010s. See A. Thomas, *review of Velvet Retro: Postsocialist Nostalgia and the Politics of Heroism in Czech Popular Culture”*, by Veronika Pehe, *Slavic Review* 81, no. 1 (2022): 245–46; and V. Pehe, *Velvet Retro: Postsocialist Nostalgia and the Politics of Heroism in Czech Popular Culture* (New York: Berghahn Books, 2020).

2. V. Pehe, “The Wild 1990s: ‘Transformation Nostalgia’ among the Czech Student Generation of 1989,” *East Central Europe* 46, no. 1 (2019): 111–34, at 113. A notable exception is James Krapfl’s excellent volume, *Revolution with a Human Face: Politics, Culture, and Community in Czechoslovakia, 1989–1992* (Ithaca, NY: Cornell University Press, 2013).

3. Exhibition homepage, Ústav pro studium totalitních režimů, https://www.ustrcr.cz/data/pdf/vystavy/my-to-nevzdali/demonstrace-pametnici.pdf (accessed 10 October 2022).

4. Ibid. The “Několik vět” petition of 1989 was actually composed by a collective of authors, not just Havel.

5. Picture taken by the author. The text is reproduced on the website of the Institute for the Study of Totalitarian Regimes (Ústav pro studium totalitních režimů, ÚSTR) (ibid.), but the exhibit mentioned on this website is undated and the design is slightly different from the exhibit I saw on Wenceslas Square in 2009. Moreover, Pavel Žáček has disappeared from this new description, which could be an indication that this new panel was produced after he had been dismissed as director of ÚSTR in 2010.

6. See M. MacDonagh-Pajerová, *Vezměte s sebou květinu! Deníky a vzpomínky z let 1980–1990* (Prague: Prostor, 2014), as well as her own description of her position provided during oral history interviews in 1996 and 1997 in M. Vaněk and M. Otáhal, *Sto studentských revolucí: Studenti v období pádu komunismu – Životospisná vyprávění* (Prague: Karolinum, 2019), 604–16.

7. Český rozhlas 1—Radiožurnál, *Radiofórum*, 19 November 1999. All translations by the author.

8. Ibid.

9. Ibid. “Sherwood” is the nickname of a park outside the Prague central railway station, which had the reputation after 1989 of being an insecure place, full of drug dealers, pickpockets, and unsavory characters.

10. Ibid.

11. Ibid.

12. Ibid.

13. Ibid.

14. Ibid. In 1989, during the mass demonstrations of the Velvet Revolution, hundreds of thousands of people shook their keys in the air to signify to the leaders that it was time for them to relinquish power and go home.

15. Ibid.

16. Ibid.

17. Ibid.

18. Ibid.

19. Ibid.

20. M. Blaive, “An Impossible Reconciliation: National (Heroic) Memory and Individual Identity in the Czech Republic,” in “L’histoire à l’école en Europe de l’Est, ed. V. Behr and E. Tartakowsky, thematic cluster, *Passés futurs 9 (2021)*, https://www.politika.io/fr/article/an-impossible-reconciliation.

21. M. Vaněk and P. Mücke, *Velvet Revolutions: An Oral History of Czech Society* (Oxford: Oxford University Press, 2016), 193. Vaněk and Mücke are here quoting Bohumír Balajka, a cooperative farmer born in 1947.

22. Ibid., 195.

23. See M. Blaive, “Codeword ‘Criminal’: Moral Remembrance in National Memory Politics,” in *CBEES* *2020 State of the Region Report: Constructions and Instrumentalization of the Past; A Comparative Study on Memory Management in the Region*, ed. Ninna Mörner (Stockholm: Södertörn University, 2021), 106–14.

24. See Pehe, “The Wild 1990s,” 116.

25. Český rozhlas 1—Radiožurnál, *Radiofórum*.

26. See K. Williams, “Czechoslovakia 1990–1992,” in *Security Intelligence Services in New Democracies: The Czech Republic, Slovakia, and Romania*, ed. K. Williams and D. Deletant (New York: Palgrave, 2001), 55–82.

27. For a demonstration of how egalitarianism was then at the core of Czech identity, see L. Holy, *The Little Czech and the Great Czech Nation*: National Identity and the Post-Communist Social Transformation (Cambridge: Cambridge University Press, 1995), 72–73.

28. M. Viewegh, *Báječná léta s Klausem* (Brno: Petrov, 2002), 27–29. This volume is more than a novel, it is a political essay and personal testimony—and an excellent source for the study of everyday life. It documents the disappointment of the author and his family, but also of large swathes of Czech society, with what they perceived as a ruthless capitalistic mentality that took hold after 1989.

29. Jan Čulík described this unethical practice, in an important talk at the Teplá cloister in 1995, to an assembly of major Czech intellectuals of the time debating the post-1989 transformation. See “Vzdělanci v dnešní České republice,” Teplá, 15 October 1995, *Britské listy*, http://www.britskelisty.cz/9803/19980327c.html#02. Čulík then recounted being fired from Radio Free Europe for attempting to prepare this report on Premier Klaus in !Jan Čulík: Nejsem komunista,” interview by Muriel Blaive for *Britské listy*, 28 October 2022, https://www.youtube.com/watch?v=InoZUHxfZqw (8:20–9:58).

30. See “Czech President Vaclav Klaus Faces Treason Charge,” BBC, 4 March 2013, https://www.bbc.com/news/world-europe-21660234.

31. Český rozhlas 1—Radiožurnál, *Radiofórum*.

32. O. Neumann and L. Tvarůžková, “Existuje ‘generace 17. listopadu’? Kdo vlastně jsou a co chtějí tvůrci výzvy Děkujeme, odejděte!” *Lidové noviny*, 29 November 1999, 3.

33. M. Pajerová, interview by M. Schulz, *Sněží*, Česká televize, 7 December 1999.

34. T. Brzobohatý, “Odejděte, po 10 letech!” *Týden*, 22 November 1999, 22.

35. Ibid.

36. E. Tabery, “Zklamaní a pobouření” Oslavy revoluce zajímají svět a zlobí ústavní činitele,” *Respekt*, 22 November 1999, 5.

37. “Autoři výzvy Děkujeme, odejděte!” *Lidové noviny*, 29 November 1999, 3.

38. Tabery, “Zklamaní a pobouření”.

39. P. Mrázková and K. Kačírková, “Aktéři 17. listopadu jsou na revoluci hrdí,” *Lidové noviny*, 17 November 1999, 2.

40. Ibid.

41. J. Grohová and Z. Janečková, “Desetitisíce demonstrantů žádaly politiky: Odejděte,” *Mladá fronta dnes*, 4 December 1999, 1.

42. I. Potřebová, “Máme právo pískat, když politici hrají falešně: Rozhovor s Igorem Chaunem, jedním z autorů výzvy Děkujeme, odejděte!” *Plzeňský deník*, 3 December 1999, 13.

43. Grohová and Janečková, “Desetitisíce demonstrantů žádaly politiky.”

44. P. Kudrna, “Podpora výzvy ‘Děkujeme, odejděte!’” **Zprávy* (15:00), Český rozhlas—Radiožurnál*, 4 December 1999.

45. E. Tabery, “Spolky, a možná i partaje,” *Respekt*, 3 December 2000, 4.

46. Potřebová, “Máme právo pískat, když politici hrají falešně.

47. “Autoři výzvy Děkujeme, odejděte!”

48. P. Polanská, “Vznik strany z výzvy ‘Děkujeme, odejděte!’ se odkládá,” *Zemské noviny*, 13 December 1999, 2.

49. “Výzva Děkujeme, odejděte! se nepřeměnila ve stranu,” *idnes.cz*, 11 December 1999, https://www.idnes.cz/zpravy/domaci/vyzva-dekujeme-odejdete-se-nepremenila-ve-stranu.A_991211_143139_domaci_jkl.

50. Polanská, “Vznik strany z výzvy.”

51. Ibid.

52. Tabery, “Spolky, a možná i partaje.”

53. See, for instance, V. Havel’s 1978 essay *The Power of the Powerless*, trans. P. Wilson, in “The Power of the Powerless Today,” ed. J. Krapfl and B. J. Falk, special issue, *East European Politics and Societies* 32, no. 2 (2018): 353–408. For the period after 1989, see V. Havel, *L’amour et la vérité doivent triompher de la haine et du mensonge* (La Tour d’Aigues: l’Aube, 1990); and V. Havel, *1992 & 1993* (Prague: Paseka, 1994).

54. “Co by se mělo v ČR změnit,” *Lidové noviny*, 29 November 1999, 2.

55. Vaněk and Otáhal, *Sto studentských revolucí*, 611.

56. Ibid.

57. Potřebová, “Máme právo pískat, když politici hrají falešně.

58. Pajerová, interview by Schulz.

59. P. Jindra, “Deset let poté na FF UK,” *Haló noviny*, 18 November 1999, 6.

60. “We are not like them!” (meaning like the Communists) was one of the slogans of the Velvet Revolution chanted by demonstrators. See Krapfl, *Revolution with a Human Face*, 29.

61. Jindra, “Deset let poté na FF UK.”

62. “Zachovejme demokracii: Úvaha; Za deset let se vše nezvládne,” *Rokycanský deník*, 2 December 1999, 17.

63. See Act on the Institute for the Study of Totalitarian Regimes and the Security Services Archive, 181/2007 Coll., 8 June 2007, https://www.ustrcr.cz/data/pdf/normy/act181-2007.pdf.

64. See M. Blaive, *Une déstalinisation manquée: Tchécoslovaquie 1956* (Brussels: Complexe, 2005), 85–86.

65. I have analyzed this phenomenon in M. Blaive, “Nostalgia and the Collective Memory of Communism in the Czech Republic: Lessons from an Oral History Study of the Last Pioneers,” in *Nostalgia on the Move*, ed. M. Slavković and M. Đorgović (Belgrade: Museum of Yugoslavia, 2017), 62–74.

66. E. Tabery, “Děkujeme, přicházejí: Výbuch občanské nespokojenosti přivedl na scénu i nejmladší generaci,” *Respekt*, 6 December 1999, 5.

67. See Tabery, “Spolky, a možná i partaje.”

68. See Blaive, “Nostalgia and the Collective Memory.”

